# Supplementary material for: Inter-regional disparities in emergency department utilization among critically ill patients: A nationwide study from South Korea
Source: PLoS One. 2025 Dec 9;20(12):e0338520. doi: 10.1371/journal.pone.0338520 (PMC12688155; doi:10.1371/journal.pone.0338520)
Supplement: Attachment — Submitted filename: 09232025 PLOS ONE_reviewer answer.docx [file pone.0338520.s001.docx]

Review Comments to the Author

We sincerely appreciate the thoughtful and constructive comments provided on our manuscript. We carefully reviewed all suggestions and revised the manuscript accordingly. Below, we provide a detailed response to each comment, with references to the specific changes made in the revised text.

**Reviewer #1:** 
**1. Introduction**: The readers are slowly introduced to the importance of the topic just before the purpose of the study is presented.
Literature Review/Conceptual Framework: There is neither a thorough literature review nor a conceptual framework to justify the hypothesis. I recommend that the authors choose one of the two for a better foundation/justification of their hypothesis.
Also, given that your hypotheses are not even known, it is hard to tell what your Dependent is and what independent and controls are. This could be addressed in a sub-section in your method section

Answer: We expanded the literature review and clarified the conceptual framework, adding relevant Korean and international references to strengthen the justification. Importantly, the newly added references are directly relevant to our topic: Korean studies (Park et al., Oh et al., Huh et al.) empirically analyzed nationwide EMS utilization patterns and determinants of patient flows, while international frameworks (Hsuan et al., Andersen) provide conceptual bases for understanding equity and healthcare utilization. These references not only strengthen the background but also provide both empirical and theoretical support for our study.

(Line 73-80)

“Despite this initiative, there has been a lack of empirical studies examining whether critically ill patients are indeed receiving care within their designated region. Previous studies have demonstrated regional disparities in EMS utilization and patient transfer patterns in Korea, classifying service areas into inflow-, mixed-, and outflow-types based on patient movement indices [8–10]. Internationally, the network equity-quality model has been proposed to conceptualize hospital transfer networks and their implications for care equity [11]. In addition, healthcare utilization has often been analyzed using Andersen’s behavioral model, which highlights the roles of predisposing, enabling, and need factors in shaping access to care [12].”

8. Park SK, Lee SH. Pattern of Regional Emergency Medical Service Utilization and Its Determinants. J Korean Soc Emerg Med. 2005;16(1):128-136.

9. Oh M, Lee JH, Jeon BU, Jeong TO, Heo T, Lee S. Usage patterns of emergency medical services in Korea: analysis of patient flow. Chin Med J (Engl). 2019 Feb 5;132(3):259-268. <https://doi.org/10.1097/CM9.0000000000000062>.

10. Young Jin Huh, Han Na Lee, Se Hyung Kim, Mi Ra Oh, Sung Min Lee. The association between demographic, economic, and medical resource factors and the inflow and outflow of critically ill emergency patients by medical service districts in South Korea. Signa Vitae. 2024. 20(10);62-71. <https://doi.org/10.22514/sv.2024.127>.

11. Hsuan C, Carr BG, Vanness D, Wang Y, Leslie DL, Dunham E, Rogowski JA. A Conceptual Framework for Optimizing the Equity of Hospital-Based Emergency Care: The Structure of Hospital Transfer Networks. Milbank Q. 2023 Mar;101(1):74-125. <https://doi.org/10.1111/1468-0009.12609>.

12. Andersen RM. Revisiting the behavioral model and access to medical care: does it matter? *J Health Soc Behav*. 1995;36(1):1–10. <https://doi.org/10.2307/2137284>.

**2. Methods**: It would be helpful to the reader to clarify what you mean by other for route of visit.

**2.1.** Line 104: Route of visit included direct visit, transfer from another facility, or other
(Answer)

According to the classification system of the National Emergency Department Information System (NEDIS) in Korea, patient visit routes to the emergency department are categorized into several predefined groups, such as direct visit, transfer from another medical institution, and referral from the outpatient department of the same institution. Patients whose routes of visit do not fall into these standard categories are classified as *“other.”* Therefore, in our study, the “other” category represents cases with atypical or unclassified visit routes that are not included in the above standard pathways. We have revised the manuscript to clarify this definition in the Methods section.

(Line 115-117)

“Route of visit included direct visit, transfer from another facility, or other. The ‘other’ category included visit routes that did not fit into the predefined classifications, such as atypical or unclassified cases.”

**2.2.** While the logistic regression in appropriate for your regression analysis, combining your logistic regression with a marginal effect will enhance your results. With the logistic regression you can only tell whether the odds are higher or lower. With the marginal effect in addition to the odds you will be able to give the percentage by which these odds are higher or lower. This will make it more quantifiable and enhance readability and comprehension for readers. Particularly those that may not be very familiar with your topic and methodology.
It will be important to report the specific statistical analysis you are reporting in your results.

(Answer)

We sincerely thank the reviewers for highlighting the limitations of our initial statistical methods and for suggesting appropriate alternatives. We conducted mixed-effects logistic regression, modified Poisson regression, and calculated average marginal effects (AMEs). Table 4 now presents ORs, aRRs, and AMEs in parallel for more comprehensive interpretation (Results, Table 4). In response, we have revised our analysis as follows. Also, in the revised manuscript, we have combined the previous Tables 5–7 into a single, reorganized Table 5 for clarity and conciseness.

(Line 135-145)

“To identify factors influencing inter-regional utilization, multivariable logistic regression analysis was performed. Because patients were clustered within the same EMS regions, we applied a mixed-effects logistic regression model with EMS region included as a random intercept to account for the clustering structure. For sensitivity analysis, we additionally fitted a standard logistic regression model and calculated cluster-robust standard errors at the EMS region level. Furthermore, given that the prevalence of inter-regional utilization in our study was relatively high (approximately 22%), and odds ratios (ORs) may overestimate the effect size, we performed modified Poisson regression to estimate adjusted risk ratios (aRRs). The results are now presented in parallel as ORs, average marginal effects (AMEs), and aRRs, including 95% confidence intervals (CIs). Some AMEs did not reach statistical significance and should be interpreted cautiously as complementary measures.”

(Line 211-233)

“We examined factors influencing inter-regional utilization among critically ill emergency patients using ORs, aRRs, and AMEs (Table 4). Male patients exhibited higher odds of inter-regional utilization compared with females (OR 1.08, 95% CI: 1.08–1.10; aRRs 1.07, 95% CI: 1.04–1.09). However, the AME indicated that the absolute probability of inter-regional use was on average 8.3 percentage points lower among men than women, highlighting a divergence between relative and absolute measures. This discrepancy underscores the importance of interpreting AMEs alongside ORs and aRRs to capture both relative and absolute perspectives. Age was also associated with inter-regional movement. Patients aged 50–64 years showed the highest predicted probability (AME +3.8%p, 95% CI: –0.23 to 7.89), while those aged 19–34 years also had elevated odds (OR 1.33, 95% CI: 1.30–1.35; aRRs 1.24, 95% CI: 1.10–1.40). Insurance type demonstrated a strong association. Compared with those covered by other insurance, patients with Medical Aid had a significantly lower likelihood of inter-regional utilization (OR 0.64, 95% CI: 0.62–0.67; aRRs 0.74, 95% CI: 0.67–0.82; AME –5.9%p, 95% CI: –7.73 to –3.99). Arrival-related factors also played an important role. Patients transferred from another hospital were more likely to receive care outside their EMS region than those presenting directly (OR 1.20, 95% CI: 1.16–1.24; AME +4.9%p, 95% CI: 2.90–6.80). Similarly, transport by ambulance (AME +2.5%p, 95% CI: 1.17–3.88) or private vehicle (AME +2.4%p, 95% CI: 1.17–3.54) was associated with a greater probability of inter-regional utilization compared with other transport modes. Clinical severity and outcomes showed more nuanced associations. Patients triaged as KTAS 1–2 had elevated odds of inter-regional use (OR 1.21, 95% CI: 1.19–1.24; aRRs 1.19, 95% CI: 0.99–1.44), although AMEs did not demonstrate significant differences. Patients ultimately transferred to other hospitals also had a higher likelihood of inter-regional utilization (OR 1.25, 95% CI: 1.17–1.32; AME +3.0%p, 95% CI: –0.43 to 6.47).”

**Table 4** Determinants of inter-regional utilization among critically ill emergency patients

| Variables | OR (95% CI) | aRRs (95% CI) | AME (%p, 95% CI) |
| --- | --- | --- | --- |
| Gender |  |  |  |
| Male | 1.08 (1.08, 1.10) | 1.07 (1.04, 1.09) | -8.29 (-12.44, -4.15) |
| Female | Ref | Ref | Ref |
| Age (yrs) |  |  |  |
| 0–18 | 0.99 (0.86, 1.02) | 1.10 (0.92, 1.33) | 1.60 (-2.62, 5.83) |
| 19–34 | 1.33 (1.30, 1.35) | 1.24 (1.10, 1.40) | 2.06 (-1.79, 5.90) |
| 35–49 | 1.19 (1.70, 1.22) | 1.12 (1.04, 1.22) | 1.65 (-1.69, 4.98) |
| 50–64 | 1.17 (1.15, 1.19) | 1.12 (1.06, 1.18) | 3.83 (-0.23, 7.89) |
| ≥65 | Ref | Ref | Ref |
| Type of ED |  |  |  |
| REMC | 1.25 (1.23, 1.27) | 0.91 (0.70, 1.18) | 1.31 (-1.86, 4.47) |
| LEMC | Ref | Ref | Ref |
| Insurance type |  |  |  |
| National health insurance | 0.82 (0.80, 0.84) | 0.96 (0.86, 1.07) | -0.99 (-3.38, 1.4) |
| Medical aid | 0.64 (0.62, 0.67) | 0.74 (0.67, 0.82) | -5.86 (-7.73, -3.99) |
| Other | Ref | Ref | Ref |
| Illness condition |  |  |  |
| Disease | 0.90 (0.89, 0.92) | 1.04 (0.89, 1.21) | 1.40 (0.84, 1.95) |
| Non, disease | Ref | Ref | Ref |
| Route of arrival |  |  |  |
| Direct visit | 0.64 (0.62, 0.66) | 0.70 (0.64, 0.76) | 2.11 (2.29, 6.52) |
| Transfer from another hospital | 1.20 (1.16, 1.24) | 1.06 (0.90, 1.24) | 4.85 (2.90, 6.80) |
| Other | Ref | Ref | Ref |
| Transportation |  |  |  |
| Ambulance | 1.12 (1.07, 1.25) | 1.10 (0.90, 1.35) | 2.52 (1.17, 3.88) |
| Private vehicle | 1.04 (0.98, 1.10) | 1.08 (0.92, 1.27) | 2.35 (1.17, 3.54) |
| Other | Ref | Ref | Ref |
| Triage |  |  |  |
| KTAS 1, 2 | 1.21 (1.19, 1.24) | 1.19 (0.99, 1.44) | -2.14 (-8.03, 3.75) |
| KTAS 3 | 1.11 (1.09, 1.13) | 1.07 (0.91, 1.25) | 0.76 (-2.53, 3.13) |
| KTAS 4, 5 | Ref | Ref | Ref |
| Final care outcome |  |  |  |
| Discharge | 1.02 (0.96, 1.08) | 1. 01 (0.89, 1.16) | 0.30 (-2.53, 3.13) |
| Transfer | 1.25 (1.17, 1.32) | 1.14 (0.99, 1.32) | 3.02 (-0.43, 6.47) |
| Death | 1.11 (1.04, 1.18) | 1.06 (0.93, 1.21) | 1.23 (-1.78, 4.24) |
| Other | Ref | Ref | Ref |

OR: Odds ratio, CI: Confidence interval, aRR: Adjusted risk ratio, AME: Average marginal effect, ED: Emergency department, REMC: Regional emergency medical center, LEMC: Local emergency medical center, KTAS: Korean triage and acuity scale.

(Line 244-250)

“As shown in the Table 5, the factors associated with inter-regional utilization differed across regions. In Seoul–Dongnam, higher odds were observed among male patients, those in the middle-aged group (50–64 years), and patients with higher acuity (KTAS 1–3). In contrast, in Daegu, younger patients (≤49 years) and those transferred or arriving by private vehicle showed markedly higher odds. In Gwangju, visits to regional emergency medical centers (REMCs) and transferred patients were strongly associated with higher odds. Notably, medical aid beneficiaries consistently exhibited lower odds across all regions.”

**Table 5** Determinants of inter, regional utilization among critically ill emergency patients: Seoul Southeast, Gwangju, and Daegu EMS regions

| Variables | OR (95% CI) | | |
| --- | --- | --- | --- |
|  | Seoul Southeast | Gwangju | Daegu |
| Gender |  |  |  |
| Male | 1.08 (1.05, 1.12) | 1.14 (1.07, 1.21) | 0.96 (0.91, 1.01) |
| Female | Ref | Ref | Ref |
| Age (yrs) |  |  |  |
| 0–18 | 0.93 (0.86, 0.99) | 1.26 (1.08, 1.47) | 1.44 (1.29, 1.62) |
| 19–34 | 0.96 (0.90, 1.02) | 1.14 (1.01, 1.29) | 1.69 (1.54, 1.84) |
| 35–49 | 1.01 (0.95, 1.06) | 1.03 (0.93, 1.15) | 1.32 (1.21, 1.44) |
| 50–64 | 1.14 (1.09, 1.19) | 1.11 (1.03, 1.20) | 1.08 (1.01, 1.16) |
| ≥65 | Ref | Ref | Ref |
| Type of ED |  |  |  |
| REMC | 1.21 (1.15, 1.28) | 1.70 (1.57, 1.84) | 0.94 (0.89, 0.99) |
| LEMC | Ref | Ref | Ref |
| Insurance type |  |  |  |
| National health insurance | 0.95 (0.87, 1.04) | 1.14 (0.98, 1.31) | 0.89 (0.77, 1.03) |
| Medical aid | 0.83 (0.74, 0.92) | 0.82 (0.69, 0.99) | 0.52 (0.44, 0.62) |
| Other | Ref | Ref | Ref |
| Illness condition |  |  |  |
| Disease | 1.24 (1.18, 1.30) | 0.98 (0.91, 1.07) | 0.93 (0.86, 1.00) |
| Non, disease | Ref | Ref | Ref |
| Route of arrival |  |  |  |
| Direct visit | 0.45 (0.42, 0.49) | 0.41 (0.35, 0.48) | 0.44 (0.40, 0.49) |
| Transfer from another hospital | 0.74 (0.68, 0.80) | 1.51 (1.29, 1.76) | 1.21 (1.08, 1.35) |
| Other | Ref | Ref | Ref |
| Transportation |  |  |  |
| Ambulance | 0.71 (0.58, 0.86) | 1.08 (0.89, 1.32) | 1.35 (1.03, 1.78) |
| Private vehicle | 0.87 (0.72, 1.05) | 1.09 (0.90, 1.32) | 1.52 (1.16, 1.98) |
| Other | Ref | Ref | Ref |
| Triage |  |  |  |
| KTAS 1, 2 | 1.49 (1.41, 1.58) | 1.10 (0.96, 1.26) | 1.08 (0.95, 1.22) |
| KTAS 3 | 1.43 (1.35, 1.50) | 1.15 (1.02, 1.30) | 1.03 (0.92, 1.16) |
| KTAS 4, 5 | Ref | Ref | Ref |
| Final care outcome |  |  |  |
| Discharge | 0.95 (0.74, 1.22) | 1. 27 (0.90, 1.78) | 1. 76 (1.09, 2.84) |
| Transfer | 0.89 (0.69, 1.15) | 1.43 (1.01, 2.02) | 1.67 (1.03, 2.71) |
| Death | 1.00 (0.78, 1.30) | 1.41 (1.01, 2.06) | 1.61 (0.99, 2.61) |
| Other | Ref | Ref | Ref |

OR: Odds ratio, CI: Confidence interval, ED: Emergency department, REMC: Regional emergency medical center, LEMC: Local emergency medical center, KTAS: Korean triage and acuity scale.

**2.3.** It will be important to explain why the use of both Wilcoxon-Mann-Whitney test and the logistic regression.

(Answer)

Thank you for this insightful comment. We used both the Wilcoxon–Mann–Whitney test and logistic regression because these methods address different aspects of our data. The Wilcoxon–Mann–Whitney test was applied to compare the distribution of continuous variables that were not normally distributed, providing a robust non-parametric comparison between groups. Logistic regression, on the other hand, was employed to identify independent associations between variables and the outcome after adjusting for potential confounders. We have clarified this rationale in the revised Methods section to improve transparency and readability.

(Line 132-134)

“Because ED and hospital LOS were not normally distributed, the non-parametric Mann–Whitney U test was used for comparisons, with results expressed as median, the first quartile (Q1), and the third quartile (Q3).”

**3. Discussion.** Why not contrast the listed regions beds per 1,000 people to that of Seoul and Gyeonggi (line 244-246).

(Answer)

We appreciate this valuable suggestion. In the revised manuscript, we added a comparison of the number of hospital beds per 1,000 people in the listed regions with those in Seoul (8.8) and Gyeonggi (9.9). This addition provides further context regarding the relative healthcare resource distribution across regions. [14]

|  | (Beds) | (Beds per 1,000 people) |
| --- | --- | --- |
| **Total** | **685,636** | **13.2** |
| Seoul | 84,678 | **8.8** |
| Busan | **69,644** | **20.5** |
| Daegu | **38,792** | **16.0** |
| Incheon | **32,694** | **11.1** |
| Gwangju | **33,912** | **23.4** |
| Daejeon | **23,125** | **15.8** |
| Ulsan | **15,068** | **13.3** |
| **Sejong** | **1,715** | **4.8** |
| Gyeonggi | **132,418** | **9.9** |
| Gangwon | **16,977** | **11.0** |
| Chungbuk | **20,959** | **13.1** |
| Chungnam | **28,127** | **13.3** |
| Jeonbuk | **37,069** | **20.5** |
| Jeonnam | **39,675** | **21.4** |
| Gyeongbuk | **42,972** | **16.3** |
| Gyeongnam | **62,984** | **18.9** |
| Jeju | **4,827** | **7.2** |
|  |  |  |

Reference 14.

5th national health and medical survey [Internet]. Sejong: National Health Insurance Service; 2022 [cited 2022 July 15]. Available from: <https://www.mohw.go.kr/board.es?mid=a10411010200&bid=0019&act=view&list_no=372195&tag=&nPage=1>.

**(Line 309-314)**

“According to the National Health and Medical Care Statistics Survey, while the total number of hospital beds is concentrated in Seoul and Gyeonggi, the number of beds per 1,000 people is higher in non-metropolitan areas, including Gwangju (21.4), Jeonnam (21.4), Busan (20.5), Jeonbuk (20.5), Gyeongnam (18.9), and Gyeongbuk (16.3). Although hospital beds are concentrated in metropolitan regions, the number of beds per 1,000 residents is higher in many non-metropolitan areas [14].”

**Reviewer #2:** Overall:
The study uses a large national dataset and shows important disparities. The question is relevant. However, the analysis is limited to one year (2021, COVID period) and policy conclusions must be more cautious.

**Major comments**

**1. Causality**: The cross-sectional design and methods (chi-square, Mann–Whitney U, logistic regression) can only show associations, not causation. Policy recommendations should be phrased more carefully.
(Answer)

We revised wording to emphasize associations only, not causality, and framed policy implications cautiously.

(Line 305-306)

“Although this association cannot be interpreted as causal, it raises concerns that delays in care may adversely affect outcomes.”

(Line 319-321)

“While these associations cannot be interpreted causally, they raise concerns that delays in definitive care may adversely affect outcomes.”

(Line 343-344)

“Finally, this study was based on a single year of cross-sectional data, restricting our ability to evaluate temporal trends or causal relationships.”

**2. Time context**: Only 2021 data are analyzed. Please discuss that later policy changes (2022–2025) may already have influenced patient flows.
(Answer)

We appreciate this valuable comment. Our analysis was limited to NEDIS data from 2021, and therefore does not capture subsequent policy changes or system reforms implemented after 2022. We agree that such changes, including the initiatives outlined in the 4th National Emergency Medical Plan (2023–2027), may already be influencing patient transfer patterns and regional utilization. To address this, we have added a statement in the Discussion acknowledging this temporal limitation and highlighting the need for future research incorporating more recent data to evaluate the impact of these policies on patient flows.

(Line 343-347)

“Finally, this study was based on a single year of cross-sectional data, restricting our ability to evaluate temporal trends or causal relationships. Moreover, subsequent policy initiatives and system reforms (2022–2025), including the 4th National Emergency Medical Plan (2023–2027), may already be affecting inter-regional patient flows, which could not be captured in our analysis.”

**3. Age grouping**: The 19–64 group is too broad. More detailed age categories or sensitivity analysis would be stronger.

(Answer)

We refined age groups in Table 3 (19–34, 35–49, 50–64, > 65 years) and revised the Results and Discussion to reflect these subgroup analyses. This provided greater granularity in describing age-related differences.

**Table 3** Comparison of general characteristics of critically ill emergency patients by inter-regional utilization status

| Variable | Total N (%) | |  | Inter-regional Utilization | | | | | *P*-value |
| --- | --- | --- | --- | --- | --- | --- | --- | --- | --- |
|  |  |  |  | Yes | |  | No | |  |
| Total* | 741,701 | (100.0) |  | 160,700 | (21.7) |  | 581,001 | (78.3) |  |
| Gender |  |  |  |  |  |  |  |  | <0.001 |
| Male | 409,537 | (55.2) |  | 91,759 | (57.1) |  | 317,778 | (54.7) |  |
| Female | 332,164 | (44.8) |  | 68,941 | (42.9) |  | 263,223 | (45.3) |  |
| Age (yrs) |  |  |  |  |  |  |  |  | <0.001 |
| 0–18 | 36,363 | (4.9) |  | 7,963 | (5.0) |  | 28,400 | (4.9) ^†^ |  |
| 19–34 | 67,708 | (9.1) |  | 16,275 | (10.1) |  | 51,433 | (8.9) ^†^ |  |
| 35–48 | 89,480 | (12.1) |  | 19,941 | (12.4) |  | 69,539 | (12.0) ^†^ |  |
| 49–64 | 174,580 | (23.5) |  | 39,281 | (24.4) |  | 135,299 | (23.3) ^†^ |  |
| ≥65 | 373,570 | (50.4) |  | 77,240 | (48.1) |  | 296,330 | (51.0) |  |
| Type of ED |  |  |  |  |  |  |  |  | <0.001 |
| REMC | 295,516 | (39.8) |  | 62,823 | (39.1) |  | 232,693 | (40.1) |  |
| LEMC | 446,185 | (60.2) |  | 97,877 | (60.9) |  | 348,308 | (59.9) |  |
| Insurance type |  |  |  |  |  |  |  |  | <0.001 |
| National health insurance | 621,485 | (83.8) |  | 137,343 | (85.5) ^†^ |  | 484,142 | (83.3) |  |
| Medical aid | 64,273 | (8.7) |  | 11,058 | (6.9) ^†^ |  | 53,215 | (9.2) |  |
| Other | 55,943 | (7.5) |  | 12,299 | (7.7) ^†^ |  | 43,644 | (7.5) |  |
| Illness condition |  |  |  |  |  |  |  |  | <0.001 |
| Disease | 534,268 | (72.0) |  | 118,192 | (73.5) |  | 416,076 | (71.6) |  |
| Non-disease | 207,433 | (28.0) |  | 42,508 | (26.5) |  | 164,925 | (28.4) |  |
| Route of arrival |  |  |  |  |  |  |  |  | <0.001 |
| Direct visit | 538,741 | (72.6) |  | 103,025 | (64.1) |  | 435,716 | (75.0) |  |
| Transfer from another hospital | 173,504 | (23.4) |  | 49,908 | (31.1) |  | 123,596 | (21.3) |  |
| Other | 29,456 | (4.0) |  | 7,767 | (4.8) |  | 21,689 | (3.7) |  |
| Transportation |  |  |  |  |  |  |  |  | <0.001 |
| Ambulance | 382,277 | (51.5) ^†^ |  | 83,566 | (52.0) |  | 298,711 | (51.4) ^†^ |  |
| Private vehicle | 351,739 | (47.4) ^†^ |  | 75,497 | (47.0) |  | 276,242 | (47.5) ^†^ |  |
| Other | 7,685 | (1.0) ^†^ |  | 1,637 | (1.0) |  | 6,048 | (1.0) ^†^ |  |
| Triage |  |  |  |  |  |  |  |  | <0.001 |
| KTAS 1-2 | 185,737 | (25.0) ^†^ |  | 44,331 | (27.6) |  | 141,406 | (24.3) ^†^ |  |
| KTAS 3 | 432,023 | (58.2) ^†^ |  | 92,433 | (57.5) |  | 339,590 | (58.4) ^†^ |  |
| KTAS 4-5 | 123,941 | (16.7) ^†^ |  | 23,936 | (14.9) |  | 100,005 | (17.2) ^†^ |  |
| Final care outcome |  |  |  |  |  |  |  |  | <0.001 |
| Discharge | 579,300 | (78.1) |  | 122,359 | (76.1) |  | 456,941 | (78.6) ^†^ |  |
| Transfer | 99,028 | (13.4) |  | 24,147 | (15.0) |  | 74,881 | (12.9) ^†^ |  |
| Death | 56,637 | (7.6) |  | 12,797 | (8.0) |  | 43,840 | (7.5) ^†^ |  |
| Other | 6,736 | (0.9) |  | 1,397 | (0.9) |  | 5,339 | (0.9) ^†^ |  |
| ED LOS (min) | 267(144-495) | |  | 305(160-581) | |  | 258(141-474) | | <0.001 |
| H LOS (day) | 7.2(3.7-14.8) | |  | 7.7(3.8-15.8) | |  | 7.0(3.7-14.7) | | <0.001 |
| Values are presented as patients (%) or median (the first quartile - the third quartile). The * is for row percentage and the others are for column percentage. ^†^ The sums of proportions are not equal to 100% due to rounding. ED: Emergency department, REMC: Regional emergency medical center, LEMC: Local emergency medical center, KTAS: Korean triage and acuity scale, LOS: Length of stay, H: Hospital | | | | | | | | | |

**4. Regression model**: Patients are nested in EMS regions, but clustering is not considered. Mixed-effects models or robust SEs should be added. Odds ratios may also exaggerate effects at 22 % prevalence; marginal effects or robust Poisson regression would be helpful.

(Answer)

We sincerely thank the reviewers for highlighting the limitations of our initial statistical methods and for suggesting appropriate alternatives. We conducted mixed-effects logistic regression, modified Poisson regression, and calculated average marginal effects (AMEs). Table 4 now presents ORs, aRRs, and AMEs in parallel for more comprehensive interpretation (Results, Table 4). In response, we have revised our analysis as follows. Also, in the revised manuscript, we have combined the previous Tables 5–7 into a single, reorganized Table 5 for clarity and conciseness.

(Line 135-145)

**Method**

“To identify factors influencing inter-regional utilization, multivariable logistic regression analysis was performed. Because patients were clustered within the same EMS regions, we applied a mixed-effects logistic regression model with EMS region included as a random intercept to account for the clustering structure. For sensitivity analysis, we additionally fitted a standard logistic regression model and calculated cluster-robust standard errors at the EMS region level. Furthermore, given that the prevalence of inter-regional utilization in our study was relatively high (approximately 22%), and odds ratios (ORs) may overestimate the effect size, we performed modified Poisson regression to estimate adjusted risk ratios (aRRs). The results are now presented in parallel as ORs, average marginal effects (AMEs), and aRRs, including 95% confidence intervals (CIs). Some AMEs did not reach statistical significance and should be interpreted cautiously as complementary measures.”

**Results**

(Line 211-233)

“We examined factors influencing inter-regional utilization among critically ill emergency patients using ORs, aRRs, and AMEs (Table 4). Male patients exhibited higher odds of inter-regional utilization compared with females (OR 1.08, 95% CI: 1.08–1.10; aRRs 1.07, 95% CI: 1.04–1.09). However, the AME indicated that the absolute probability of inter-regional use was on average 8.3 percentage points lower among men than women, highlighting a divergence between relative and absolute measures. This discrepancy underscores the importance of interpreting AMEs alongside ORs and aRRs to capture both relative and absolute perspectives. Age was also associated with inter-regional movement. Patients aged 50–64 years showed the highest predicted probability (AME +3.8%p, 95% CI: –0.23 to 7.89), while those aged 19–34 years also had elevated odds (OR 1.33, 95% CI: 1.30–1.35; aRRs 1.24, 95% CI: 1.10–1.40). Insurance type demonstrated a strong association. Compared with those covered by other insurance, patients with Medical Aid had a significantly lower likelihood of inter-regional utilization (OR 0.64, 95% CI: 0.62–0.67; aRRs 0.74, 95% CI: 0.67–0.82; AME –5.9%p, 95% CI: –7.73 to –3.99). Arrival-related factors also played an important role. Patients transferred from another hospital were more likely to receive care outside their EMS region than those presenting directly (OR 1.20, 95% CI: 1.16–1.24; AME +4.9%p, 95% CI: 2.90–6.80). Similarly, transport by ambulance (AME +2.5%p, 95% CI: 1.17–3.88) or private vehicle (AME +2.4%p, 95% CI: 1.17–3.54) was associated with a greater probability of inter-regional utilization compared with other transport modes. Clinical severity and outcomes showed more nuanced associations. Patients triaged as KTAS 1–2 had elevated odds of inter-regional use (OR 1.21, 95% CI: 1.19–1.24; aRRs 1.19, 95% CI: 0.99–1.44), although AMEs did not demonstrate significant differences. Patients ultimately transferred to other hospitals also had a higher likelihood of inter-regional utilization (OR 1.25, 95% CI: 1.17–1.32; AME +3.0%p, 95% CI: –0.43 to 6.47).”

**Table 4** Determinants of inter, regional utilization among critically ill emergency patients

| Variables | OR (95% CI) | aRRs (95% CI) | AME (%p, 95% CI) |
| --- | --- | --- | --- |
| Gender |  |  |  |
| Male | 1.08 (1.08, 1.10) | 1.07 (1.04, 1.09) | -8.29 (-12.44, -4.15) |
| Female | Ref | Ref | Ref |
| Age (yrs) |  |  |  |
| 0–18 | 0.99 (0.86, 1.02) | 1.10 (0.92, 1.33) | 1.60 (-2.62, 5.83) |
| 19–34 | 1.33 (1.30, 1.35) | 1.24 (1.10, 1.40) | 2.06 (-1.79, 5.90) |
| 35–49 | 1.19 (1.70, 1.22) | 1.12 (1.04, 1.22) | 1.65 (-1.69, 4.98) |
| 50–64 | 1.17 (1.15, 1.19) | 1.12 (1.06, 1.18) | 3.83 (-0.23, 7.89) |
| ≥65 | Ref | Ref | Ref |
| Type of ED |  |  |  |
| REMC | 1.25 (1.23, 1.27) | 0.91 (0.70, 1.18) | 1.31 (-1.86, 4.47) |
| LEMC | Ref | Ref | Ref |
| Insurance type |  |  |  |
| National health insurance | 0.82 (0.80, 0.84) | 0.96 (0.86, 1.07) | -0.99 (-3.38, 1.4) |
| Medical aid | 0.64 (0.62, 0.67) | 0.74 (0.67, 0.82) | -5.86 (-7.73, -3.99) |
| Other | Ref | Ref | Ref |
| Illness condition |  |  |  |
| Disease | 0.90 (0.89, 0.92) | 1.04 (0.89, 1.21) | 1.40 (0.84, 1.95) |
| Non, disease | Ref | Ref | Ref |
| Route of arrival |  |  |  |
| Direct visit | 0.64 (0.62, 0.66) | 0.70 (0.64, 0.76) | 2.11 (2.29, 6.52) |
| Transfer from another hospital | 1.20 (1.16, 1.24) | 1.06 (0.90, 1.24) | 4.85 (2.90, 6.80) |
| Other | Ref | Ref | Ref |
| Transportation |  |  |  |
| Ambulance | 1.12 (1.07, 1.25) | 1.10 (0.90, 1.35) | 2.52 (1.17, 3.88) |
| Private vehicle | 1.04 (0.98, 1.10) | 1.08 (0.92, 1.27) | 2.35 (1.17, 3.54) |
| Other | Ref | Ref | Ref |
| Triage |  |  |  |
| KTAS 1, 2 | 1.21 (1.19, 1.24) | 1.19 (0.99, 1.44) | -2.14 (-8.03, 3.75) |
| KTAS 3 | 1.11 (1.09, 1.13) | 1.07 (0.91, 1.25) | 0.76 (-2.53, 3.13) |
| KTAS 4, 5 | Ref | Ref | Ref |
| Final care outcome |  |  |  |
| Discharge | 1.02 (0.96, 1.08) | 1. 01 (0.89, 1.16) | 0.30 (-2.53, 3.13) |
| Transfer | 1.25 (1.17, 1.32) | 1.14 (0.99, 1.32) | 3.02 (-0.43, 6.47) |
| Death | 1.11 (1.04, 1.18) | 1.06 (0.93, 1.21) | 1.23 (-1.78, 4.24) |
| Other | Ref | Ref | Ref |

OR: Odds ratio, CI: Confidence interval, aRR: Adjusted risk ratio, AME: Average marginal effect, ED: Emergency department, REMC: Regional emergency medical center, LEMC: Local emergency medical center, KTAS: Korean triage and acuity scale.

(Line 244-250)

“As shown in the Table 5, the factors associated with inter-regional utilization differed across regions. In Seoul–Dongnam, higher odds were observed among male patients, those in the middle-aged group (50–64 years), and patients with higher acuity (KTAS 1–3). In contrast, in Daegu, younger patients (≤49 years) and those transferred or arriving by private vehicle showed markedly higher odds. In Gwangju, visits to regional emergency medical centers (REMCs) and transferred patients were strongly associated with higher odds. Notably, medical aid beneficiaries consistently exhibited lower odds across all regions.”

**Table 5** Determinants of inter, regional utilization among critically ill emergency patients: Seoul Southeast, Gwangju, and Daegu EMS regions

| Variables | OR (95% CI) | | |
| --- | --- | --- | --- |
|  | Seoul Southeast | Gwangju | Daegu |
| Gender |  |  |  |
| Male | 1.08 (1.05, 1.12) | 1.14 (1.07, 1.21) | 0.96 (0.91, 1.01) |
| Female | Ref | Ref | Ref |
| Age (yrs) |  |  |  |
| 0–18 | 0.93 (0.86, 0.99) | 1.26 (1.08, 1.47) | 1.44 (1.29, 1.62) |
| 19–34 | 0.96 (0.90, 1.02) | 1.14 (1.01, 1.29) | 1.69 (1.54, 1.84) |
| 35–49 | 1.01 (0.95, 1.06) | 1.03 (0.93, 1.15) | 1.32 (1.21, 1.44) |
| 50–64 | 1.14 (1.09, 1.19) | 1.11 (1.03, 1.20) | 1.08 (1.01, 1.16) |
| ≥65 | Ref | Ref | Ref |
| Type of ED |  |  |  |
| REMC | 1.21 (1.15, 1.28) | 1.70 (1.57, 1.84) | 0.94 (0.89, 0.99) |
| LEMC | Ref | Ref | Ref |
| Insurance type |  |  |  |
| National health insurance | 0.95 (0.87, 1.04) | 1.14 (0.98, 1.31) | 0.89 (0.77, 1.03) |
| Medical aid | 0.83 (0.74, 0.92) | 0.82 (0.69, 0.99) | 0.52 (0.44, 0.62) |
| Other | Ref | Ref | Ref |
| Illness condition |  |  |  |
| Disease | 1.24 (1.18, 1.30) | 0.98 (0.91, 1.07) | 0.93 (0.86, 1.00) |
| Non, disease | Ref | Ref | Ref |
| Route of arrival |  |  |  |
| Direct visit | 0.45 (0.42, 0.49) | 0.41 (0.35, 0.48) | 0.44 (0.40, 0.49) |
| Transfer from another hospital | 0.74 (0.68, 0.80) | 1.51 (1.29, 1.76) | 1.21 (1.08, 1.35) |
| Other | Ref | Ref | Ref |
| Transportation |  |  |  |
| Ambulance | 0.71 (0.58, 0.86) | 1.08 (0.89, 1.32) | 1.35 (1.03, 1.78) |
| Private vehicle | 0.87 (0.72, 1.05) | 1.09 (0.90, 1.32) | 1.52 (1.16, 1.98) |
| Other | Ref | Ref | Ref |
| Triage |  |  |  |
| KTAS 1, 2 | 1.49 (1.41, 1.58) | 1.10 (0.96, 1.26) | 1.08 (0.95, 1.22) |
| KTAS 3 | 1.43 (1.35, 1.50) | 1.15 (1.02, 1.30) | 1.03 (0.92, 1.16) |
| KTAS 4, 5 | Ref | Ref | Ref |
| Final care outcome |  |  |  |
| Discharge | 0.95 (0.74, 1.22) | 1. 27 (0.90, 1.78) | 1. 76 (1.09, 2.84) |
| Transfer | 0.89 (0.69, 1.15) | 1.43 (1.01, 2.02) | 1.67 (1.03, 2.71) |
| Death | 1.00 (0.78, 1.30) | 1.41 (1.01, 2.06) | 1.61 (0.99, 2.61) |
| Other | Ref | Ref | Ref |

OR: Odds ratio, CI: Confidence interval, ED: Emergency department, REMC: Regional emergency medical center, LEMC: Local emergency medical center, KTAS: Korean triage and acuity scale.

**5. Model specification**: Some variables (triage, final outcome) may be mediators. Please clarify model structure and provide diagnostics (multicollinearity, fit, AUC, calibration).

(Answer)

We thank the reviewer for this valuable comment. Our primary objective was to identify patient- and system-level factors associated with inter-regional utilization rather than to build a predictive model or establish causal pathways. We acknowledge that some variables, such as triage severity and final clinical outcomes, may act as mediators rather than independent predictors. To clarify this, we emphasized in the Methods and Discussion that our model was designed as an explanatory, association-based framework rather than a causal model.

Regarding model diagnostics, we assessed multicollinearity using variance inflation factors (VIFs). All VIF values were below 5, indicating no significant collinearity. Given the explanatory nature of our analysis, we did not conduct additional predictive model diagnostics.

(Line 144-145)

“To evaluate model validity, multicollinearity was assessed using variance inflation factors (VIFs).”

(Line 232-233)

All VIF values were below 5, indicating no significant multicollinearity among independent variables.

**6. LOS interpretation**: Differences in ED and hospital LOS are descriptive only, not causal. Please present carefully.

(Answer)

We thank the reviewer for this valuable comment. In line with the suggestion, we revised the *Results* and *Discussion* to ensure clarity. In the Results, we present differences in ED and hospital length of stay (LOS) descriptively without causal interpretation. These revisions emphasize that our findings reflect associations rather than causal relationships.

(Line 304-306)

“High-acuity patients who received inter-regional care also had longer ED stays. Although this association cannot be interpreted as causal, it raises concerns that delays in care may adversely affect outcomes.”

(Line 318-321)

“Patients who received inter-regional care had significantly longer ED and hospital length of stay compared with those treated within their residential region. While these associations cannot be interpreted causally, they raise concerns that delays in definitive care may adversely affect outcomes.”

**Minor comments**

**1. Check tables for possible errors (Table 3 numbers, Table 6 CI).**

Table 3:

(Answer)

We thank the reviewer for this valuable comment. The column percentages for each variable were rounded to the second decimal place. As a result, the sum of the percentages may occasionally appear as 100.1% or 99.9% instead of exactly 100%. This minor discrepancy arises from the rounding process and does not affect the validity or interpretation of the analysis.

Table 5 CI:

Table has been re-modified / 0.93 (0.80–1.09)

**2. Use consistent terminology** (“out-of-region” vs. “inter-regional”).

(Answer)

We revised the manuscript to consistently use the term “inter-regional utilization” throughout the text, tables, and figures.

**3. Add units in tables and STROBE checklist reference**.

(Answer)

We have added units (*N %,* minutes, day) to the tables and explicitly referenced the STROBE checklist by including a statement in the Methods section.

(Line 156-157)

“This study adhered to the STROBE reporting guideline [13].”
