# Supplementary material for: Inter-regional disparities in emergency department utilization among critically ill patients: A nationwide study from South Korea
Source: PLoS One. 2025 Dec 9;20(12):e0338520. doi: 10.1371/journal.pone.0338520 (PMC12688155; doi:10.1371/journal.pone.0338520)
Supplement: Attachment — Submitted filename: 11082025 PLOS ONE_reviewer answer-2nd.docx [file pone.0338520.s002.docx]

Review Comments to the Author

We sincerely appreciate the thoughtful and constructive comments provided on our manuscript. We carefully reviewed all suggestions and revised the manuscript accordingly. Below, we provide a detailed response to each comment, with references to the specific changes made in the revised text.

**Reviewer #1:** 
**1. The authors addressed my concerns and made the paper stronger. The paper brings an important contribution to extant knowledge.**

Answer: Thank you for your thoughtful review.

**Reviewer #2:**

1. The current age categories (0–18, 19–64, ≥65) are not adequate and obscure key heterogeneity. Comparable studies use finer bands (e.g., 0–4, 5–12, 13–18; 19–44, 45–64, 65–74, 75–84, ≥85). Please re-bin and re-run primary analyses (or model age continuously with splines) and update tables/results accordingly.

**Answer:**
Thank you for this important suggestion. We fully agree that age effects may be heterogeneous, particularly among pediatric and older adult subgroups.

In response, we re-classified age into eight standard epidemiologic groups
(0–4, 5–12, 13–18, 19–44, 45–64, 65–74, 75–84, and ≥85 years),
and re-estimated all primary models (OR, aRR, and AME).

This refinement revealed more granular and clinically coherent patterns of inter-regional utilization. As reflected in the updated Tables 3–5:

- Younger patients (0–4 and 5–12 years) and
  middle-aged adults (19–44 and 45–64 years) had *higher* odds of inter-regional utilization.
- Older adults (≥75 years)—particularly those ≥85 years—were *least* likely to receive care outside their EMS region.
- Re-estimation of all models produced modest shifts in effect sizes, largely due to redistribution across narrowed age bands.

We revised the Abstracts, Methods, Results, and Discussion to reflect the updated classification and interpretation.

**Abstracts**

**Line 32-45:** although their absolute probability was significantly lower (AME –8.29%p, 95% CI: –12.42 to –4.16), reflecting differences between relative and absolute measures. Adults aged 19–44 years (OR 1.35, 95% CI: 1.32–1.39) and 45–64 years (OR 1.24, 95% CI: 1.21–1.27) showed higher odds of inter-regional utilization, although corresponding AMEs were not statistically significant. Among older adults, the 65–74-year group showed a non-significant decrease in absolute probability (AME –0.90%p, 95% CI: –3.39 to 1.58), whereas the 75–84-year group demonstrated a significant reduction (AME –5.78%p, 95% CI: –7.64 to –3.92). Pediatric patients also exhibited elevated odds of inter-regional use. Medical Aid beneficiaries had lower relative odds (OR 0.65, 95% CI: 0.63–0.67), and AMEs showed no statistically significant absolute differences. Inter-hospital transfer (OR 1.20, 95% CI: 1.16–1.24) was associated with higher inter-regional use, with a statistically significant increase in absolute probability (AME +3.74%p, 95% CI: 0.86–6.63). Arrival by ambulance or private vehicle showed positive but non-significant AMEs. High-acuity patients (KTAS 1–2) and those transferred as their final disposition also demonstrated higher relative odds of inter-regional utilization.

**Methods**

**Line 117-119**: Sex was categorized as male or female, and age was grouped as 0–4 years, 5–12 years, 13–18 years, 19–44 years, 45–64 years, 65–74 years, 75–84 years, and ≥85 years to capture more granular age-related variation in inter-regional emergency care utilization.

**Results**

**Line 203-205**: Age-specific analysis revealed a bimodal pattern: higher proportions among younger (0–4 and 5–12 years) and middle-aged adults (19–44 and 45–64 years), whereas patients aged ≥85 years were least likely to receive care outside their EMS region (p<0.001).

**Line 227-232** Age was also associated with inter-regional movement. Adults aged 19–44 years (OR 1.35, 95% CI: 1.32–1.39) and 45–64 years (OR 1.24, 95% CI: 1.21–1.27) had higher odds than those aged ≥85 years, although their AMEs were not statistically significant. Among older adults, the 65–74-year group showed a non-significant decrease in absolute probability (AME –0.90%p, 95% CI: –3.39 to 1.58), whereas the 75–84-year group demonstrated a significant reduction (AME –5.78%p, 95% CI: –7.64 to –3.92). Pediatric patients also exhibited elevated odds of inter-regional use.

**Line 235-250**: Insurance type showed a strong association. Medical Aid beneficiaries had a significantly lower likelihood of inter-regional utilization across all effect measures (OR 0.64, 95% CI: 0.62–0.67; aRR 0.74, 95% CI: 0.67–0.82; AME –5.90%p, 95% CI: –7.73 to –3.99). National Health Insurance beneficiaries showed a nonsignificant positive AME.

Patients with disease-related conditions had a higher absolute probability of inter-regional use (AME +1.40%p, 95% CI: 0.84–1.95). Arrival-related factors also played an important role. Inter-hospital transfer was associated with higher relative odds (OR 1.20, 95% CI: 1.16–1.24) and a statistically significant increase in absolute probability (AME +3.74%p, 95% CI: 0.86–6.63). Direct visits showed an even larger absolute probability difference (+5.39%p).

Regarding transportation, ambulance (AME +2.91%p, 95% CI: –0.73 to 6.54) and private vehicle arrivals (AME +0.78%p, 95% CI: –0.87 to 2.42) displayed positive but non-significant AMEs. High-acuity patients (KTAS 1–2) had higher relative odds (OR 1.21, 95% CI: 1.18–1.23), although corresponding AMEs were non-significant. Patients whose final disposition was transfer also showed elevated odds (OR 1.25, 95% CI: 1.17–1.33), with a small, non-significant increase in absolute probability. All VIF values were below 5, indicating no multicollinearity.

**Line 262 - 271**: In Seoul–Southeast, higher odds were observed among male patients, a broad range of middle- and older-aged adults (45–74 years), and patients with higher acuity (KTAS 1–3). In Gwangju, pediatric patients demonstrated notably high odds across all age groups under 18 years, and visits to regional emergency medical centers (REMCs) showed the strongest association with inter-regional utilization. Patients transferred from another hospital also had significantly elevated odds. In Daegu, the highest odds were observed among younger adults (19–44 years), with elevated risks also present among pediatric groups. Additionally, both ambulance and private vehicle arrivals were associated with higher odds of inter-regional utilization, suggesting more active inter-regional movement in response to capacity or resource limitations.

**Discussion**

**Line 289-292**: In Daegu, younger patients and those transferred or arriving by private or ambulance transport had higher odds, whereas in Gwangju, REMC visits and inter-hospital transfers were strongly associated with inter-regional utilization. Pediatric patients also showed elevated odds in Gwangju, reflecting gaps in regional pediatric emergency capacity.

**Line 296-300:** Multivariable analyses revealed notable differences between relative and absolute effect measures. Male patients showed higher adjusted odds of inter-regional utilization; however, AMEs demonstrated that men had significantly lower absolute predicted probabilities than women. This divergence is consistent with methodological literature indicating that ORs and AMEs may not align when baseline probabilities differ, underscoring the need to present multiple effect measures for accurate interpretation [20-22].

**Line 304-311**: Younger and middle-aged adults—particularly those in the 19–44 and 45–64 year groups—showed higher rates of inter-regional utilization, whereas older adults were less likely to receive care outside their EMS region. In our analysis, AMEs for younger groups were positive but not statistically significant, whereas older age groups demonstrated significant decreases in absolute probability, indicating reduced mobility and greater dependence on local services among older adults. Notably, the reduction was statistically significant only in the 75–84-year group, whereas the decrease observed in the 65–74-year group was not significant.

**Line 323-326**: Our AME findings further showed significantly lower absolute probabilities among Medical Aid beneficiaries, reinforcing the interpretation that financial and social vulnerability constrain inter-regional mobility. This pattern underscores how financial and social vulnerability can influence healthcare-seeking behavior and contribute to disparities in emergency care access.

**Line 332-336**: although their AMEs were not statistically significant, this suggests that relative differences in transport patterns do not necessarily translate to meaningful absolute increases in utilization. Although inter-hospital transfers showed higher relative odds, direct visits demonstrated a larger absolute probability of inter-regional use, indicating that patient-driven decisions and system-driven transfers may operate through different pathways.

**Line 340-345**: Patients who received inter-regional care had significantly longer ED and hospital lengths of stay compared with those treated within their residential region. Although these associations cannot be interpreted causally, they raise concerns that delays in timely definitive care may adversely affect outcomes. Supporting this, recent Korean studies have shown that EDs implementing structured length-of-stay management systems for critically ill patients achieved lower in-hospital mortality and shorter hospitalization durations [20, 23].

**Line 363-364**: regional disparities in emergency care infrastructure—rather than preference alone—appear to be the predominant drivers.

**Table 3** Comparison of general characteristics of critically ill emergency patients by inter-regional utilization status

| Variable | Total N (%) | |  | Inter-regional Utilization | | | | | *P*-value |
| --- | --- | --- | --- | --- | --- | --- | --- | --- | --- |
|  |  |  |  | Yes | |  | No | |  |
| Total* | 741,701 | (100.0) |  | 160,700 | (21.7) |  | 581,001 | (78.3) |  |
| Gender |  |  |  |  |  |  |  |  | <0.001 |
| Male | 409,537 | (55.2) |  | 91,759 | (57.1) |  | 317,778 | (54.7) |  |
| Female | 332,164 | (44.8) |  | 68,941 | (42.9) |  | 263,223 | (45.3) |  |
| Age (yrs) |  |  |  |  |  |  |  |  | <0.001 |
| 0–4 | 15,079 | (2.0) |  | 3,801 | (2.4) |  | 11,278 | (1.9) |  |
| 5–12 | 9,477 | (1.3) |  | 2,030 | (1.3) |  | 7,447 | (1.3) |  |
| 13–18 | 11,807 | (1.6) |  | 2,132 | (1.3) |  | 9,675 | (1.7) |  |
| 19–44 | 122,044 | (16.5) |  | 28,551 | (17.8) |  | 93,493 | (16.1) |  |
| 45–64 | 209,724 | (28.3) |  | 46,946 | (29.2) |  | 162,778 | (28.0) |  |
| 65–74 | 134,263 | (18.1) |  | 29,651 | (18.5) |  | 104,612 | (18.0) |  |
| 75–84 | 160,196 | (21.6) |  | 32,375 | (20.1) |  | 127,821 | (22.0) |  |
| ≥85 | 79,111 | (10.7) |  | 15,214 | (9.5) |  | 63,897 | (11.0) |  |
| Type of ED |  |  |  |  |  |  |  |  | <0.001 |
| REMC | 295,516 | (39.8) |  | 62,823 | (39.1) |  | 232,693 | (40.1) |  |
| LEMC | 446,185 | (60.2) |  | 97,877 | (60.9) |  | 348,308 | (59.9) |  |
| Insurance type |  |  |  |  |  |  |  |  | <0.001 |
| National health insurance | 621,485 | (83.8) |  | 137,343 | (85.5) ^†^ |  | 484,142 | (83.3) |  |
| Medical aid | 64,273 | (8.7) |  | 11,058 | (6.9) ^†^ |  | 53,215 | (9.2) |  |
| Other | 55,943 | (7.5) |  | 12,299 | (7.7) ^†^ |  | 43,644 | (7.5) |  |
| Illness condition |  |  |  |  |  |  |  |  | <0.001 |
| Disease | 534,268 | (72.0) |  | 118,192 | (73.5) |  | 416,076 | (71.6) |  |
| Non-disease | 207,433 | (28.0) |  | 42,508 | (26.5) |  | 164,925 | (28.4) |  |
| Route of arrival |  |  |  |  |  |  |  |  | <0.001 |
| Direct visit | 538,741 | (72.6) |  | 103,025 | (64.1) |  | 435,716 | (75.0) |  |
| Transfer from another hospital | 173,504 | (23.4) |  | 49,908 | (31.1) |  | 123,596 | (21.3) |  |
| Other | 29,456 | (4.0) |  | 7,767 | (4.8) |  | 21,689 | (3.7) |  |
| Transportation |  |  |  |  |  |  |  |  | <0.001 |
| Ambulance | 382,277 | (51.5) ^†^ |  | 83,566 | (52.0) |  | 298,711 | (51.4) ^†^ |  |
| Private vehicle | 351,739 | (47.4) ^†^ |  | 75,497 | (47.0) |  | 276,242 | (47.5) ^†^ |  |
| Other | 7,685 | (1.0) ^†^ |  | 1,637 | (1.0) |  | 6,048 | (1.0) ^†^ |  |
| Triage |  |  |  |  |  |  |  |  | <0.001 |
| KTAS 1-2 | 185,737 | (25.0) ^†^ |  | 44,331 | (27.6) |  | 141,406 | (24.3) ^†^ |  |
| KTAS 3 | 432,023 | (58.2) ^†^ |  | 92,433 | (57.5) |  | 339,590 | (58.4) ^†^ |  |
| KTAS 4-5 | 123,941 | (16.7) ^†^ |  | 23,936 | (14.9) |  | 100,005 | (17.2) ^†^ |  |
| Final care outcome |  |  |  |  |  |  |  |  | <0.001 |
| Discharge | 579,300 | (78.1) |  | 122,359 | (76.1) |  | 456,941 | (78.6) ^†^ |  |
| Transfer | 99,028 | (13.4) |  | 24,147 | (15.0) |  | 74,881 | (12.9) ^†^ |  |
| Death | 56,637 | (7.6) |  | 12,797 | (8.0) |  | 43,840 | (7.5) ^†^ |  |
| Other | 6,736 | (0.9) |  | 1,397 | (0.9) |  | 5,339 | (0.9) ^†^ |  |
| ED LOS (min) | 267 (144-495) | |  | 305 (160-581) | |  | 258 (141-474) | | <0.001 |
| H LOS (day) | 7.2 (3.7-14.8) | |  | 7.7 (3.8-15.8) | |  | 7.0(3.7-14.7) | | <0.001 |
| Values are presented as patients (%) or median (the first quartile - the third quartile). The * is for row percentage and the others are for column percentage. ^†^ The sums of proportions are not equal to 100% due to rounding. ED: Emergency department, REMC: Regional emergency medical center, LEMC: Local emergency medical center, KTAS: Korean triage and acuity scale, LOS: Length of stay, H: Hospital | | | | | | | | | |

**Table 4** Determinants of inter-regional utilization among critically ill emergency patients

| Variables | OR (95% CI) | aRR (95% CI) | AME (%p, 95% CI) |
| --- | --- | --- | --- |
| Gender |  |  |  |
| Male | 1.08 (1.07, 1.10) | 1.05 (1.03, 1.08) | -8.29 (-12.42, -4.16) |
| Female | Ref | Ref | Ref |
| Age (yrs) |  |  |  |
| 0–4 | 1.16 (1.11, 1.21) | 1.32 (1.04, 1.69) | 1.60 (-2.61, 5.80) |
| 5–12 | 1.06 (1.00, 1.12 | 1.18 (0.87, 1.59) | 2.18 (-1.56, 5.92) |
| 13–18 | 0.90 (0.85, 0.95) | 1.02 (0.80, 1.29) | 1.61 (-1.72, 4.93) |
| 19–44 | 1.35 (1.32, 1.39) | 1.29 (1.15, 1.44) | 3.70 (-0.33, 7.73) |
| 45–64 | 1.24 (1.21, 1.27) | 1.20 (1.07, 1.34) | 1.32 (-1.83, 4.47) |
| 65–74 | 1.13 (1.11, 1.16) | 1.15 (1.00, 1.34) | -0.90 (-3.39, 1.58) |
| 75–84 | 1.02 (1.00, 1.04) | 1.04 (0.96, 1.13) | -5.78 (-7.64, -3.92) |
| ≥85 | Ref | Ref | Ref |
| Type of ED |  |  |  |
| REMC | 1.25 (1.23, 1.26) | 0.90 (0.70, 1.17) | 1.14 (0.67, 1.62) |
| LEMC | Ref | Ref | Ref |
| Insurance type |  |  |  |
| National health insurance | 0.82 (0.80, 0.84) | 0.96 (0.85, 1.08) | 6.14 (-0.71, 12.99) |
| Medical aid | 0.65 (0.63, 0.67) | 0.75 (0.67, 0.83) | 3.34 (-3.66, 10.33) |
| Other | Ref | Ref | Ref |
| Illness condition |  |  |  |
| Disease | 0.90 (0.89, 0.92) | 1.04 (0.89, 1.21) | 1.40 (0.84, 1.95) |
| Non, disease | Ref | Ref | Ref |
| Route of arrival |  |  |  |
| Direct visit | 0.64 (0.62, 0.66) | 0.70 (0.64, 0.76) | 5.39 (2.83, 7.96) |
| Transfer from another hospital | 1.20 (1.16, 1.24) | 1.06 (0.90, 1.24) | 3.74 (0.86, 6.63) |
| Other | Ref | Ref | Ref |
| Transportation |  |  |  |
| Ambulance | 1.19 (1.12, 1.26) | 1.11 (0.91, 1.35) | 2.91 (-0.73, 6.54) |
| Private vehicle | 1.04 (0.98, 1.10) | 1.08 (0.92, 1.26) | 0.78 (-0.87, 2.42) |
| Other | Ref | Ref | Ref |
| Triage |  |  |  |
| KTAS 1, 2 | 1.21 (1.18, 1.23) | 1.19 (0.98, 1.43) | -2.17 (-8.05, 3.72) |
| KTAS 3 | 1.11 (1.09, 1.13) | 1.07 (0.91, 1.25) | 0.71 (-2.71, 4.13) |
| KTAS 4, 5 | Ref | Ref | Ref |
| Final care outcome |  |  |  |
| Discharge | 1.01 (0.95, 1.07) | 1. 01 (0.88, 1.15) | 0.18 (-2.59, 2.95) |
| Transfer | 1.25 (1.17, 1.33) | 1.14 (0.99, 1.32) | 3.03 (-0.48, 6.54) |
| Death | 1.12 (1.05, 1.19) | 1.07 (0.93, 1.22) | 1.36 (-1.78, 4.49) |
| Other | Ref | Ref | Ref |

OR: Odds ratio, CI: Confidence interval, aRR: Adjusted risk ratio, AME: Average marginal effect, ED: Emergency department, REMC: Regional emergency medical center, LEMC: Local emergency medical center, KTAS: Korean triage and acuity scale.

**Table 5** Determinants of inter-regional utilization among critically ill emergency patients: Seoul Southeast, Gwangju, and Daegu EMS regions

| Variables | OR (95% CI) | | |
| --- | --- | --- | --- |
|  | Seoul Southeast | Gwangju | Daegu |
| Gender |  |  |  |
| Male | 1.06 (1.02, 1.10) | 1.12 (1.05, 1.19) | 0.96 (0.91, 1.01) |
| Female | Ref | Ref | Ref |
| Age (yrs) |  |  |  |
| 0–4 | 1.22 (1.10, 1.36) | 1.65 (1.27, 2.15) | 1.75 (1.48, 2.07) |
| 5–12 | 1.13 (0.98, 1.31) | 1.43 (1.08, 1.90) | 1.72 (1.34, 2.19) |
| 13–18 | 1.06 (0.92, 1.22) | 1.18 (0.90, 1.55) | 1.38 (1.09, 1.75) |
| 19–44 | 1.19 (1.11, 1.28) | 1.20 (1.06, 1.37) | 1.88 (1.68, 2.09) |
| 45–64 | 1.41 (1.32, 1.51) | 1.22 (1.09, 1.37) | 1.20 (1.08, 1.33) |
| 65–74 | 1.41 (1.32, 1.51) | 1.17 (1.04, 1.32) | 1.18 (1.06, 1.31) |
| 75–84 | 1.20 (1.12, 1.28) | 1.11 (0.99, 1.25) | 1.15 (1.04, 1.28) |
| ≥85 | Ref | Ref | Ref |
| Type of ED |  |  |  |
| REMC | 1.22 (1.16, 1.29) | 1.70 (1.57, 1.84) | 0.95 (0.90, 1.00) |
| LEMC | Ref | Ref | Ref |
| Insurance type |  |  |  |
| National health insurance | 0.96 (0.88, 1.05) | 1.14 (0.98, 1.32) | 0.90 (0.78, 1.04) |
| Medical aid | 0.84 (0.75, 0.93) | 0.82 (0.69, 0.98) | 0.54 (0.45, 0.64) |
| Other | Ref | Ref | Ref |
| Illness condition |  |  |  |
| Disease | 1.23 (1.17, 1.29) | 0.98 (0.91, 1.07) | 0.92 (0.85, 0.99) |
| Non, disease | Ref | Ref | Ref |
| Route of arrival |  |  |  |
| Direct visit | 0.45 (0.42, 0.49) | 0.41 (0.35, 0.48) | 0.44 (0.40, 0.49) |
| Transfer from another hospital | 0.74 (0.68, 0.80) | 1.51 (1.30, 1.76) | 1.21 (1.09, 1.35) |
| Other | Ref | Ref | Ref |
| Transportation |  |  |  |
| Ambulance | 0.72 (0.59, 0.87) | 1.08 (0.89, 1.32) | 1.36 (1.03, 1.78) |
| Private vehicle | 0.87 (0.72, 1.05) | 1.08 (0.90, 1.31) | 1.50 (1.15, 1.96) |
| Other | Ref | Ref | Ref |
| Triage |  |  |  |
| KTAS 1, 2 | 1.48 (1.39, 1.57) | 1.10 (0.96, 1.25) | 1.07 (0.94, 1.22) |
| KTAS 3 | 1.42 (1.35, 1.50) | 1.15 (1.02, 1.30) | 1.03 (0.92, 1.16) |
| KTAS 4, 5 | Ref | Ref | Ref |
| Final care outcome |  |  |  |
| Discharge | 0.94 (0.73, 1.21) | 1. 26 (0.89, 1.77) | 1. 75 (1.08, 2.82) |
| Transfer | 0.90 (0.69, 1.16) | 1.42 (1.01, 2.01) | 1.68 (1.03, 2.72) |
| Death | 1.01 (0.78, 1.31) | 1.44 (1.00, 2.06) | 1.61 (0.99, 2.62) |
| Other | Ref | Ref | Ref |

OR: Odds ratio, CI: Confidence interval, ED: Emergency department, REMC: Regional emergency medical center, LEMC: Local emergency medical center, KTAS: Korean triage and acuity scale.
